# A multiverse analysis of cleaning and analyzing procedures of eye movement data during reading

**DOI:** 10.3758/s13428-025-02689-0

**Published:** 2025-05-07

**Authors:** Hayward J. Godwin, Charlotte E. Lee, Denis Drieghe

**Affiliations:** School of Psychology, University of Southampton, Highfield, Southampton, Hampshire SO17 1BJ UK

**Keywords:** Reading, Eye movements, Multiverse analysis

## Abstract

Eye movements during reading experiments involve careful cleaning of raw data into a processed format that can then be analyzed. Through the process of cleaning and analyzing these datasets, there are many decisions that researchers make. As a result, there is a wide range of possible approaches that can be taken when analyzing datasets from reading and eye movement experiments. At present, little is known regarding the consequences of these decisions and in a worst-case scenario, specific approaches to cleaning and analyzing these datasets could “create” effects that would otherwise not be present in the datasets. Here, we addressed these issues by conducting a multiverse analysis of a range of reasonable and defensible analyses that researchers in this field might conduct. We examined a total of 1,890 different data cleaning and analytic pipelines to explore how different decisions researchers make when cleaning and analyzing their data influence perhaps the most well-known effect in eye movements and reading research: the word frequency effect. More specifically, the impact on the size of the word frequency effect during sentence reading (Lee et al. *Journal of Experimental Psychology: Learning, Memory, and Cognition*, 2025) was explored. The frequency effect was found to be extremely robust and present in almost all cases, but the magnitude varied substantially, with 36% of the size of the effect being due to specific choices made during data cleaning and analysis. Recommendations for further work and greater transparency in the field are set out based on our findings.

Eye tracking is considered to be the methodological gold standard utilized for the study of reading (Rayner, 2009). Eye movements are thought to reflect cognitive processing during reading, and this assumption is corroborated by an extensive body of evidence showing how a variety of linguistic variables influence both the duration and location of eye movements. Perhaps the most well documented of these influences is the so-called frequency effect, whereby fixation durations on high-frequency words (e.g., *apple*) are shorter than those on low-frequency words (e.g., *lychee*; Inhoff & Rayner, 1986; Rayner & Duffy, 1986). As with many research methods, the analysis of eye movement behavior involves a complex pipeline through which datasets are cleaned and then analyzed by researchers both in reading and in other fields (e.g., see Godwin et al., 2021). The consequence of this is that a given researcher working on an eye movement and reading experiment has a staggering number of options to choose from when cleaning and analyzing their dataset. These options are not in any way controversial and are accepted practice in the field, despite the fact that, as discussed below, the origin of some of the accepted practices are largely unknown and not documented in terms of published empirical validations. All of this leads to a very high number of researcher degrees of freedom (Simmons et al., 2011), where researchers can make many different decisions with regard to how to clean, process, and analyze their data, coupled with a range of questions regarding what differences any cleaning procedures are making to the conclusions drawn from experiments. Here, we directly examined the impact of the cleaning and analytic decisions made by eye movement and reading researchers by conducting a multiverse analysis. This enabled us to map out the outcomes of a large set of these decisions that are routinely made—but never discussed openly—by researchers when examining the frequency effect during reading.

As mentioned above, the data processing and analytic pipeline for eye movements and reading experiments involves a series of decisions that researchers make when working with their datasets. It is important to note that some of these decisions are implicit, often reflecting the established practices in their field, which have been referred to as the “epistemic culture” of a field (Cetina, 1999), including, in its simplest form, how to set up and use an eye tracker, which eye tracker to use, and so on (for a related discussion, see Harder, 2020). This knowledge will often go beyond what is included in the manual of the manufacturer of the eye tracker, and it is not unusual for this knowledge to be passed on from more experienced members of a laboratory to more junior researchers, and/or to those with less experience using a technique, piece of equipment, or analytic method (e.g., see Darch et al., 2015).

Other decisions in the pipeline are explicit in that they are (or at least *should be*) noted in a publication deriving from a dataset. These can include decisions such as removing specific data points as outliers or deciding to remove specific participants for exhibiting poor performance, or for other reasons such as the fact that participants were not paying attention when taking part in the study. Other explicit decisions can involve the use of a specific dependent measure to analyze instead of others, a specific statistical test to use, a certain piece of software or version of that software, and so on.

Additionally, what can be an implicit decision in some studies can be very much explicit in others. For example, most recent reading research employs an EyeLink eye tracker for the registration of eye movement behavior (Eskenazi, 2024). When publications studying reading do not mention the specific algorithm that was used to group the individual samples of fixation positions into saccades and fixations, one can assume that the default algorithm implemented in the EyeLink tracker was used. However, research has shown that there is considerable confusion in the eye tracking community about the operational definition of what constitutes a fixation and a saccade (Hessels et al., 2018), and in eye tracking fields other than reading, the choice of the algorithm for determining eye movement events can be very much explicit. Likewise, in a recent review, Godwin et al. (2021) surveyed a sample of eye tracking publications and found that only 70% reported the algorithm they used for parsing fixations and saccades. Of those that reported the algorithm used, there were 11 different combinations of settings used. Clearly, then, eye movement researchers are using a wide variety of different approaches even for the most basic aspect of their work: deciding whether the eyes are fixating or not.

The many potential decisions made by researchers—as well as the combinations of those decisions that are made when working with a dataset—have been referred to as the “garden of forking paths,” with each decision made representing a fork in a path (Gelman & Loken, 2014). Especially when the choices that are made are considered defensible, the question can of course be raised whether variations in choices would produce qualitatively different outcomes. One of the ways in which this issue has been examined in the past is by recruiting many different teams of expert researchers at different laboratories and asking these different teams to analyze the same dataset to test the same hypothesis. Results of these *many analyst* studies have consistently found that researchers rarely use the same pipelines to address the same hypothesis or research question to examine the same dataset (for examples, see: Botvinik-Nezer et al., 2020; Dutilh et al., 2019; Schweinsberg et al., 2021; Silberzahn et al., 2018). In fact, what these many analyst studies have consistently found is that the approaches taken by different research teams to use the same dataset to address the same hypothesis can lead to conclusions that do not just include an effect being significant versus not significant: instead, effects can be nonsignificant for some researchers, and for others can be significant in one direction, whilst for a third group, effects can be significant *in the opposite direction.* These findings have given further support to the notion that researcher degrees of freedom enable too great a level of flexibility when analyzing datasets from psychological experiments.

For this paper, we used an alternative approach to tackle this issue of flexibility in analytic approaches by using a multiverse analysis to explore the “garden of forking paths” in eye movement and reading research (Simonsohn et al., 2020; Steegen et al., 2016). Our multiverse analysis examined the frequency effect across a large range of defensible and reasonable analyses that researchers in the field might utilize. The key terms in the previous sentence are *defensible* and *reasonable*: one should not, in an analysis of this type, create analytic pipelines that make little sense, or that researchers would not use in actual studies. For example, one should use reasonable limits for outliers as opposed to absurdly high or low limits that would not be used in practice. This is because the goal is to map out the different approaches that researchers might take in the garden of forking paths, rather than approaches that would not be reasonable to adopt. After analyzing the results from a wide range of different pipelines, a *specification curve* is produced that plots out an effect under those different pipelines. Based on the outcome of the full set of analyses, conclusions can then be drawn regarding whether an effect is stable across the entire multiverse.

Multiverse analyses have already been valuable in a wide range of different domains. They are often used when there is disagreement in the literature regarding whether a reported finding is robust. These analyses have the potential to show under which circumstances (i.e., which routes through the garden of forking paths) an effect might be significant or not. Indeed, they can also highlight that an effect is robust if it will survive diverging choices in the pipeline, as long as these choices are reasonable. The most dramatic example is, of course, when the analyses demonstrate that an effect only holds when a single or small number of analytic pipelines are used. We refer the interested reader to the work of Stafford (2018), who reported evidence of a “reverse stereotype threat” effect for female players in chess. A subsequent multiverse analysis conducted by Smerdon et al. (2020) revealed that the effects purported to be found by Stafford (2018) only held in a narrow range of conditions, and that, for most reasonable, defensible analyses, the claims by made Stafford (2018) did not hold. To put this another way, the conclusions made by Stafford (2018) only held “when the stars aligned” and were not, on the whole, reliable or robust against different data processing or analytic approaches.

Turning to the approach taken in the current study, we expected that the frequency effect would still hold across a wide range of different analytic pipelines, and this was for several reasons. First, the frequency effect has been widely replicated in many different languages by different laboratories and researchers across the globe (e.g., Kuperman et al., 2024). As such, many different pipelines have already been used alongside many conceptual replications. In fact, we chose the frequency effect for this project for the exact reason that we know it to be highly robust. However, we believe that examining a robust effect is an important first step in mapping how different data analytic pipelines can influence the size of a trusted and well-known effect in the study of eye movements and reading. This is particularly the case because, as we discuss in more detail below, some aspects of the cleaning process that researchers use for their eye movement and reading experiments are not as well validated as might be expected or is widely assumed in the field. For that reason, it is not necessarily a foregone conclusion that the frequency effect will prevail against all possible combinations of reasonable, defensible analyses. However, given the robustness of the frequency effect, we expect that the most interesting findings will relate to the impact of the data analytic pipelines on the size of the frequency effect. Future work will then be able to examine other effects that are known to be less robust (or perhaps more controversial) in the context of eye movements and reading. We return to this issue in the general discussion.

## Decision points included

We will now describe the different factors that we considered for inclusion for our multiverse analysis. We refer to each of these as a *decision point*, since they are key points where researchers need to make explicit decisions regarding what approach to take when cleaning and analyzing their datasets. There are four such decision points that we used here, and we will describe each of these in turn. Our use of these decision points is supported by a recent review conducted by Eskenazi (2024), whose work highlights that our decision points and approaches taken are indeed reported in publications and as such can be considered acceptable practice. Though it is possible to conduct a multiverse analysis that also includes different orders of cleaning and analytic steps, here we used the same order of cleaning throughout. We cleaned from the lowest level of the data (at the level of the fixations) through to the highest level of the data (at the level of participants and groups/conditions). We believe it is unlikely, and would be illogical, to change this order—for example, there would be little sense in cleaning by participant first and *after that* removing individual outlier fixations. For that reason, our ordering of cleaning and decision points remained fixed throughout.

### Decision point #1: Merging of fixations and temporal cutoffs

The first decision point for researchers examining reading and eye movement data involves how to handle very long and very short fixations. Perhaps unsurprisingly, different approaches can be used to clean very brief or very long fixations. A widely used practice in reading research is the use of a temporal cutoff for very short fixations. If fixation durations are shorter than this cutoff and are close to another, longer fixation, the shorter fixations are then merged with the longer fixation. If the very short fixation is not close to another longer fixation, the short fixation is, instead, removed. The reason for merging a very short fixation with a nearby longer fixation comes from research using the double-step paradigm (Becker & Jürgens, 1979). This paradigm established that these very brief fixations often represent cases where the eyes landed in an inappropriate location and a very fast correction is carried out, and that the duration of that short fixation is not influenced by the visual processing of the information at the inappropriate location.

Besides merging, an additional temporal cutoff can be used to remove both very short fixations (not close to another longer fixation) and unusually long fixations (which might represent tracker error or an outlier for other reasons).

To allow for these corrections to happen automatically, the software provided by EyeLink to process collected eye movement data, Data Viewer, contains a *cleaning function*. This elaborate cleaning function consists of four stages. In the first stage of the cleaning function, fixations shorter than 80 ms are merged with a longer fixation that is located within half a degree of visual angle. The second stage involves merging fixations shorter than 40 ms with longer fixations located within 1.25 degrees of visual angle. In stage three, when three fixations shorter than 140 ms are found to be within the same area of interest,^1^ they are merged to form one much longer fixation. In stage four, of the remaining fixations, those shorter than 140 ms and longer than 800 ms are removed. Finally, after all these procedures have been carried out, there is also an option to to delete all fixations that landed outside the areas of interest. However, this latter option is, by default, turned off.

Many of the values used as the default settings in the cleaning function as described above are fixed. Eskenazi (2024) tried to find the origin of these specific values and reported that some researchers believe that the defaults originate from the work of Keith Rayner, but that the exact origin and justification remain unclear. In our own personal communication with the EyeLink manufacturer (SR Research, 26/06/22), we were told that the four-stage fixation cleaning was based on “an in-house program originally used in Avital Deutsch’s lab at Hebrew University.” That is, there does not appear to be any published demonstration that these cleaning functions have been empirically validated. It is therefore vital that we both map out and understand the effects that they have on our datasets. We hope it is clear from this description that these cleaning procedures and the cutoffs used in them deserve a closer look, even though this should not be taken as a criticism of the invaluable work by pioneers in this field.

### Decision point #2: Removing trials with few fixations

The next decision point that researchers need to consider is how to clean and treat trials with very few fixations. Trials in which very few fixations are made when reading a long sentence (perhaps as few as one fixation) are believed to reflect the fact that the participant either erroneously ended a trial too soon or was not paying attention. These trials are sometimes removed by researchers since the participant is believed to have not been properly engaged when reading the stimuli.

### Decision point #3: Removal of outlier fixations

The third decision point for researchers involves how to decide whether a given fixation is an outlier, beyond the temporal cutoffs noted in the previous section. The most frequently used method in the field (Eskenazi, 2024) is based on means and standard deviation cutoffs to determine whether given fixations can be regarded as outliers. The most commonly used criteria for removing fixations are either 2.5 or 3 standard deviations away from the mean. However, we also note that researchers sometimes select the grand mean, the mean for a specific subject, or the mean for a specific subject within a specific condition, to calculate standard deviation cutoffs (e.g., Fitzsimmons et al., 2019). There is, therefore, considerable flexibility in determining outliers for fixations themselves, just as there is for the cleaning function.

### Decision point #4: Linear mixed effects models

After all of the cleaning procedures have been carried out on the eye movement data, we can reach the final decision point, and that focuses on the analyses undertaken to draw conclusions from the dataset. Linear mixed effects (LME) models are typically implemented for analyzing eye movement measures in reading experiments. However, LME models assume that the dependent variable follows a normal distribution, whereas eye movement measures often follow an ex-Gaussian distribution (e.g., Staub et al., 2010). This often raises the question whether eye movement data should be log-transformed prior to running the models. Unfortunately, this is not necessarily a perfect solution, because log transformations can have secondary effects on how variables map onto behavior (see Balota et al., 2013). Clearly, the debate around this is beyond the scope of the current paper, but, for now, what we do note is that in the literature, we see linear mixed models being reported on both untransformed and log-transformed eye movement data. Finally, a third option is using a gamma distribution for generalized linear mixed models, thereby allowing for the analysis of skewed eye movement data without transformation (Lo & Andrews, 2015). Given the very high number of linear mixed models we ran for the multiverse analysis (see below), we opted out of computational reasons to run intercept-only models thereby side-stepping the issue of determining the best way to trim the maximal random structure (Barr et al., 2013). Examining the impact of trimming strategies on effect sizes in eye movements during reading would be an exciting application of a multiverse analysis in future research.

## Decision points not included

There were a small number of potential decision points that we considered to vary within our multiverse analysis, but ultimately decided to keep fixed here. These were the choice of eye tracker, the underlying algorithm used to parse fixations and saccades, and finally the choice of dependent variable. We shall briefly outline these now and explain why they were not included as decision points in our multiverse analysis.

### Choice of eye tracker

There are many different types of eye trackers that could be used to collect data during a study of the type under investigation here. Eskenazi (2024) reported, in his review of eye movement during reading papers published in 10 top-tier journals in a time widow from 2018 to 2022, that 96% of studies collected eye movement data with an EyeLink, illustrating the dominance of this eye tracker in the reading field (similar findings have been reported in other fields that record eye movements; see Godwin et al., 2021). We therefore focused here on eye movement data that had been registered by an EyeLink eye tracker and did not consider other eye trackers.

### Parsing algorithm

The raw output of an eye tracker consists of a time stamp of when the fixation location was determined, and an *x* and *y* coordinate of the screen where the system projects the eye is looking at that specific moment in time. These samples are subsequently processed by an algorithm in which velocity and acceleration thresholds determine the onset and offset of saccades. Samples above these thresholds are considered part of a saccade, and other samples are considered part of a fixation. Although the EyeLink does allow these thresholds to be altered, and indeed recommends adjusting them for certain research areas (e.g., microsaccades), reading researchers typically do not make changes to the thresholds. With that in mind, here we did not vary the different underlying parsing algorithm settings and instead assumed that the default EyeLink parsing algorithm was used (SR Research, 2017).

### Choice of dependent variable

In the reading literature, a large number of eye movement measures have been defined and are routinely used by researchers for different purposes. These include first fixation durations (duration of the first fixation on a word of region of interest), single fixation durations (the duration of the only fixation on a word or region of interest), gaze durations (sum of the fixations on a word or region of interest before leaving the region), total fixation durations (the sum of all fixations on a word or region of interest regardless of whether these fixations were made during first-pass reading or rereading), and many more (for a review, see Rayner, 2009).

A study of eye movements in reading will typically report several eye movement measures, as they are assumed to reflect different stages in the processing of a word (Rayner, 2009). The first fixation duration is, for instance, assumed to be an “earlier” processing measure of a word compared to total fixation duration. We list the choices of dependent measures here, as this does reflect one of the “forking paths.” However, it is less relevant for our exploration of the impact of the choices made during cleaning and analyzing on the size of reported effects such as the frequency effect. For that reason, here, for our multiverse analysis, we instead focused exclusively on single fixation durations on our target words.

### Single sentences versus paragraphs

One more decision we needed to make was whether we would analyze eye movement behavior during the reading of a sentence or of a paragraph. This distinction is relevant particularly in the context of cleaning fixations. To give but one example, whether a short fixation located close to the second line of a paragraph would be merged with a longer fixation on the first line of the paragraph will have repercussions on whether subsequent fixations on the second line will count as first-pass reading. That is, if the short fixation close to the second line is not merged, it will count as the first-pass reading time of the second line, and all subsequent fixations on the second line will be considered rereading. Decisions made concerning the cleaning and analysis pipelines might therefore differentially impact reported effects in sentence versus paragraph reading. For the current investigation, we will examine the comparatively more straightforward situation of sentence reading but note the need to repeat this analysis in paragraph reading.

## Navigating the garden of forking paths: The current study

From the list of analytic decisions listed above, there are a very large number of potential combinations of different pipelines that could be used to analyze the same dataset from an eye movement during reading experiment. Any one of these could be selected by researchers as the “correct” pipeline and reported in a study, avoiding the other potential and equally viable pipelines that could have been adopted.

At this point, it is important to discuss an important predecessor to the current study which we have referenced several times already. Eskenazi (2024) carried out a detailed review and examination of the cleaning procedures for eye movement data in reading research. His examination focused on temporal cutoffs, merging methods, and outlier removal methods. In the first part of his paper, he carried out a systematic review, noted above, which found evidence of a high level of inconsistency in the reporting and application of data cleaning methods in the field. He followed this up by running three different cleaning methods on a dataset from an eye movement and reading experiment: a baseline model in which there was no merging of fixations, no removal based on temporal outliers, and no removal based on standard deviations from the subject-specific mean; a second model in which only stage 1 of the cleaning function of Data Viewer was used, where fixations shorter than 80 ms and longer than 800 ms are removed as well as fixations three standard deviations away from the subject-specific mean; and finally, a model where stages 1, 2, and 3 of the cleaning function of Data Viewer were used, and where fixations shorter than 140 ms and longer than 600 ms were removed, alongside a removal of fixations 2.5 standard deviations away from the subject-specific mean. The idea behind this approach was that the third of these models would filter out more data than the second model, which in turn would remove more than the first model.

Of particular interest for the current study is that Eskenazi (2024) reported a clear relationship between the percentage of data points removed and the size of the frequency effect: as the percentage of data points removed increased, the size of the frequency effect decreased. However, this reduction in size was also accompanied by decreased variance such that effects remained significant. In the current study, we go considerably beyond the work reported by Eskenazi (2024) by examining a much wider range of choices used in terms of data cleaning, but also by examining different types of approaches available to researchers when deciding how their linear mixed models are applied. Moreover, we use formal analytic techniques designed to truly test patterns of results over many different data processing pipelines.

## Method

### Base dataset

The dataset that we used here is derived from a study examining individual differences in skilled reading and the size of the frequency effect (Lee et al., 2025). The experiment consisted of 100 average-to-very-skilled readers who were native speakers of English without any known reading difficulties. The participants read 78 sentences that featured either a high- or a low-frequency word matched on word length. The high-frequency words had higher Zipf values (*M* = 5.28*, SD* = 0.36) than the low-frequency words (*M* = 3.22, *SD* = 0.43*)* according to the SUBTLEX -UK corpus (Van Heuven et al., 2014). All words were nouns and had a word length of on average 5.4 letters (*SD* = 0.87, range 4–9). Participants were instructed to read for comprehension and were asked occasional questions about the sentences they had just read. For further technical details on this dataset we refer to Lee et al. (2025).

### Analytic pipelines included

As part of a multiverse analysis, all reasonable combinations of analytic pipelines are run and collated. Each individual analytic pipeline is referred to as a universe. Here, each universe involved the combination of four different decision points. These were as follows:

#### Decision point #1: Merging of fixations and temporal cutoffs

We generated a total of 14 base datasets using different settings for the cleaning function in Data Viewer. These are listed in Table 1. Values were chosen to cover a wide range of possibilities that would be considered reasonable.

**Table 1:**
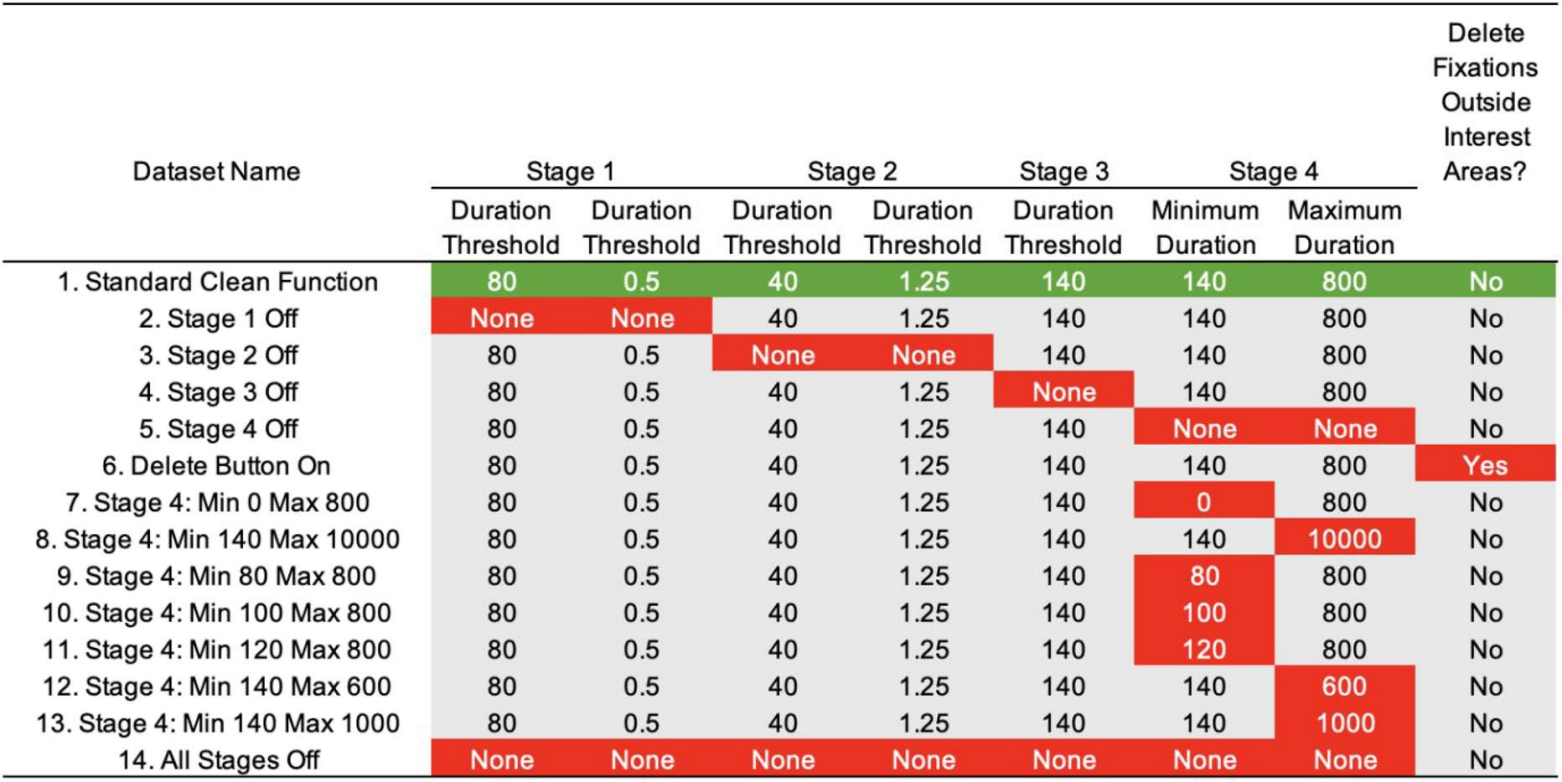
Cleaning function settings used to create 14 different datasets

Green shaded area highlights the standard cleaning function settings; cells have been shaded in red in order to highlight how each dataset diverges from the standard cleaning function settings

#### Decision point #2: Removing trials with few fixations

For this decision point in the data processing pipeline, we included trials only when they had > 0 fixations (i.e., excluding no trials based on this criterion), > 3 fixations, or > 5 fixations. As with decision point #1, values were again chosen to cover a set of possibilities that would be considered reasonable.

#### Decision point #3: Removal of outlier fixations

For this decision point, we either did not remove any trials or we removed trials based on standard deviations away the grand mean, from the mean per subject, or the mean of the subject for that specific condition. The criterion was either 2.5 or 3 standard deviations away from the selected mean. We chose these for reasons described above regarding this decision point.

#### Decision point #4: Linear mixed effects models

We ran a linear mixed model on the untransformed data, on the log-transformed data, or on the untransformed data without assuming a normal distribution (Lo & Andrews, 2015). Again, we used these three different models for reasons described above regarding this decision point.

## Results

Across the different decision points, taken together, we had a total of 1,890 universes in the empirical multiverse analysis (note that this was after the removal of combinations that were redundant or illogical, such as a decision point to not trim by participant-level means and then including a criterion such that fixations that were 2.5 standard deviations from a mean should be removed). In order to determine whether the multiverse analysis was significant, we conducted a set of permutation tests, following one of the approaches listed by Simonsohn et al. (2020). These permutation tests involved randomly shuffling the independent variable of word frequency in all of the datasets in order to remove its association with the dependent variable. We did this 500 times and then repeated the multiverse analyses on these shuffled datasets as well, effectively creating 500 multiverse analyses wherein any association between word frequency and observed fixation durations was removed.

We ran linear mixed models using the *lme4* package version 1.1–35.2 (Bates et al., 2015) on single fixation durations with word frequency entered as a categorical predictor. To reduce the processing time for the total 946,890 models (1,890 models in the empirical multiverse and then this number again multiplied 500 using the shuffled dataset) that we ran, we opted to run intercept-only models for the random factors, which were subjects and items.

### Multiverse analysis

**Fig. 1:**
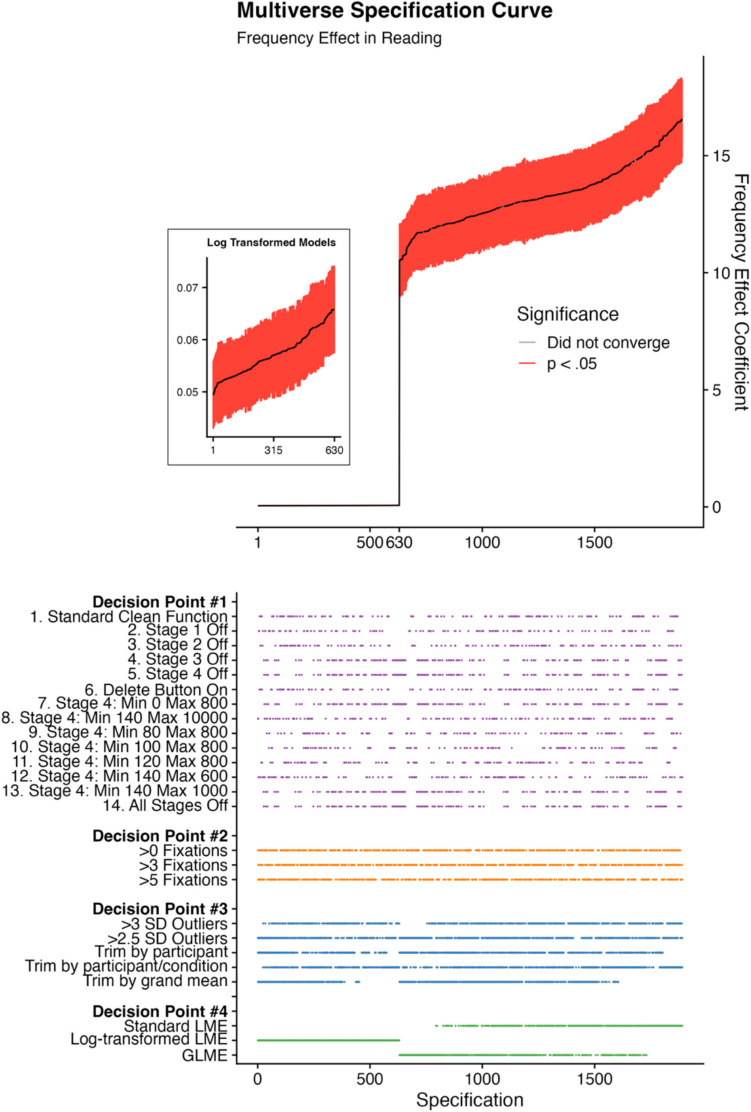
Multiverse specification curve and dashboard plots showing how the frequency effect varies based on settings in the different decision points

We undertook the multiverse analysis and present the results of this analysis in Fig. 1. The upper panel of Fig. 1 presents a specification curve. Each universe here is presented and ordered from low to high according to the size of the frequency effect coefficient in the linear mixed models. Models that showed evidence of a significant frequency effect are presented in red; those that did not converge are presented with gray dots. The inset panel of Fig. 1 presents a “zoomed-in” view of the log-transformed models, which naturally had smaller frequency effect coefficients than the non-log-transformed models. The lower panel of Fig. 1 aligns with the upper panel and presents a dashboard plot outlining the combination of decision points in the analytic processing pipeline. When a dot is present on this dashboard plot for any decision point, the corresponding universe had that decision point “active” as part of its analytic pipeline. For example, where there are points visible for the first “Model 1” row, these are present where Model 1 was used. To use another example, where dots are visible for “Trim grand mean,” these signify that for those universes where the dots are visible, the grand mean was trimmed. Where the dots are not visible, then the grand mean was not trimmed.

In a standard multiverse analysis, points in the upper panels of a visualization such as Fig. 1 would be set to one color where a given effect is significant and a different color where a given effect is not significant. Here, the frequency effect was significant at the *p* < 0.05 level in the majority of cases, and for that reason there is largely only one color present in Fig. 1. In fact, of the 1,890 models that formed part of the multiverse analysis, only 12 did not converge (~ 0.6%), and the frequency effect in the rest of the models was significant. To put this another way, in the vast majority of cases, the models converged, and when they did, they revealed evidence of a significant effect of word frequency. The 14 models that did not converge are presented as gray dots in the upper panel of Fig. 1, and were spread across the entire specification curve, with no clear evidence of them arising as a result of specific decision points in the analytic pipelines.

In order to determine whether this overall analysis was “significant,” we followed standard procedure here outlined in previous work. Within the 500 shuffled multiverse analyses, none had the same number (or greater) of significant frequency effects as in our empirical multiverse (i.e., the non-shuffled, true datasets). In such a scenario, therefore, we can conclude that the *p-*value of our multiverse was *p* < 0.002 (i.e., it is less frequent than one of the 500 shuffled repetitions of the analyses).

### How did the decision points influence the frequency effect?

We will now turn to reporting how the different approaches taken at each decision point influenced the frequency effect in our analysis. A few points are worth noting from the outset here. First, and perhaps most importantly, multiverse analyses can seem “unsatisfying” to researchers, as we often expect (or hope for) clear answers from the analyses that we run. Should we ask direct questions such as whether a given decision point makes the frequency effect larger or smaller, then it is unlikely that we will obtain a direct answer. That is because each decision point does not stand alone in these analyses. Instead, for each universe in a multiverse analysis, the combination of decisions made form an entirely unique pipeline that shifts and changes the dataset in what is typically a very unpredictable manner. For example, setting different maximum and minimum thresholds for fixation durations before they are removed and/or merged at decision point #1 will result in a change in the number of fixations per trials in some cases. That in turn can influence whether or not a trial is removed entirely from the dataset based upon there being too few fixations during that trial (decision point #2). Next, at decision point #3, a number of different approaches can be taken regarding the removal of outliers at a higher level. Once all of that is complete, we can then decide at decision point #4, which of three types of LME to use. Even small changes in the values used at each decision point can have a surprisingly large impact on the final outcome of an analysis.

Ultimately, then, we suggest that each decision point should not generally be viewed in a unidimensional manner: the end product of each universe is, of course, the result of a complex and unpredictable pipeline of data cleaning and processing steps. Because the use of cleaning functions in datasets of this type is so important, in the next section we focus on how different clean function settings influenced the frequency effect.

**Fig. 2:**
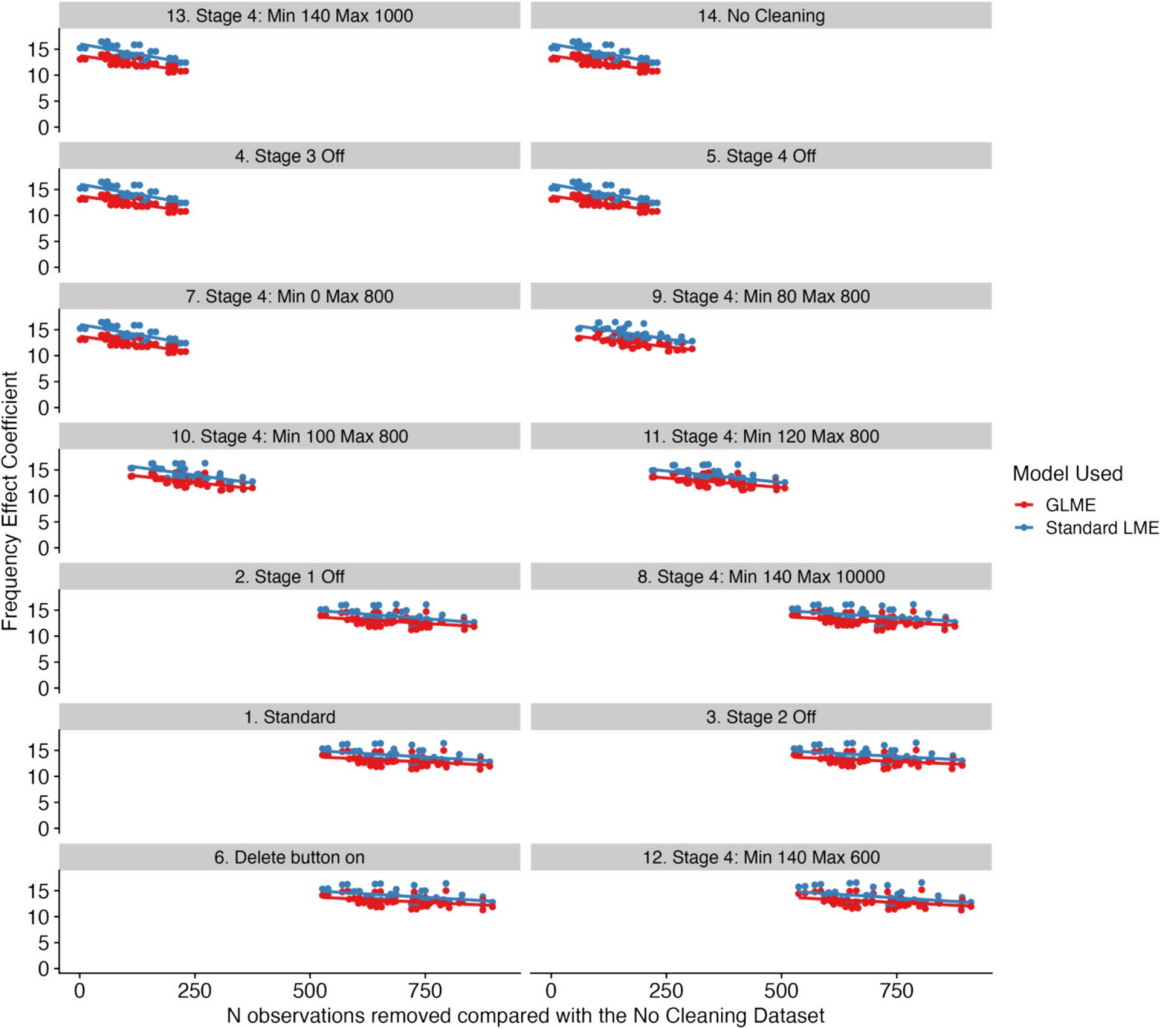
Plots comparing the number of observations removed compared to the No Cleaning dataset versus the frequency effect coefficient for all base datasets in the multiverse, for the standard LME model and the GLME model. *Note*. Lines of best fit have been added for visualization purposes only

For the remaining decision points, our interpretation of the analyses was as follows. For decision point #2, we observed no clear pattern regarding the removal of trials based on the number of fixations within them with regard to the magnitude of the frequency effect. This can be observed in Fig. 1 by the fact that the orange dots for decision point #2 are rather evenly spread across all specifications. Similarly, for decision point #3, the blue dots on the dashboard plot in Fig. 2 are evenly spread across the specifications, suggesting that no one approach necessarily increases or decreases the magnitude of the frequency effect—that is, aside from trimming by the grand mean. When trimming by the grand mean, there was some indication that the frequency effect was smaller, as evidenced by the dots in the dashboard plot in Fig. 2 clustering towards the lower end of each of the different types of LME models. On the topic of the models themselves, for decision point #4, we found the clearest pattern overall. Log-transformed LME models exhibited the smallest frequency effects (as would be expected), with generalized LME (GLME) models following soon after, and then standard LME models exhibiting the largest frequency effects in general.

### Follow-up: Comparing the base datasets

**Fig. 3:**
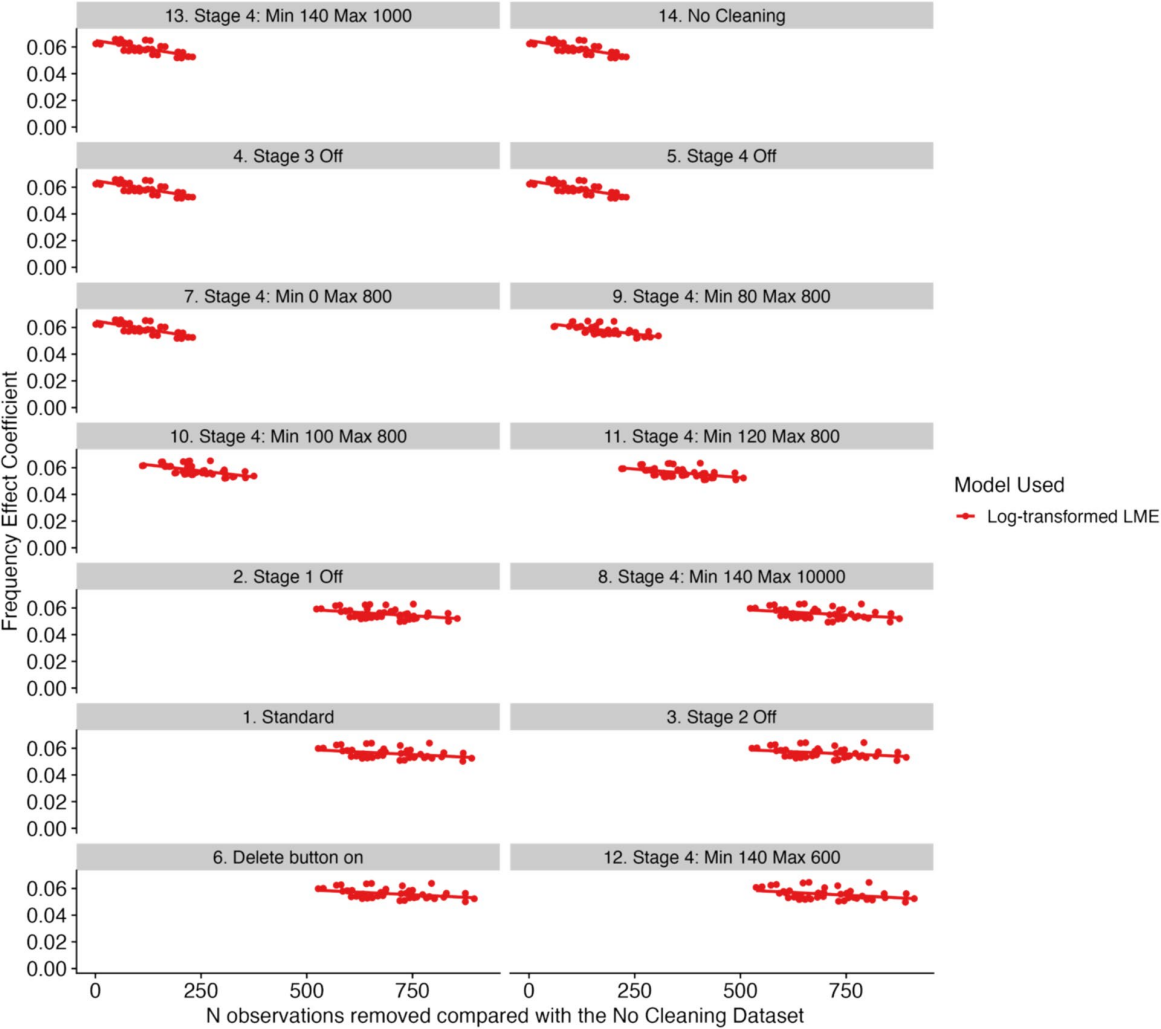
Plots comparing the number of observations removed compared to the No Cleaning dataset versus the frequency effect coefficient for all base datasets in the multiverse, for the log-transformed model. *Note*. Lines of best fit have been added for visualization purposes only

The final set of comparisons that we undertook involved a comparison of the base datasets after using the cleaning function, focusing in detail on the effects of decision point #1 in isolation. This is of interest because, as noted above, little is known regarding what differences the different options provided by the cleaning function actually lead to in practice. To gain insights into this issue, we examined the model coefficients as a function of the number of observations removed compared with the dataset that had all stages of the cleaning function turned off. We have plotted this in Fig. 2 (for the standard LME model and the GLME model) and Fig. 3 (for the log-transformed model, separated due to the far smaller coefficients than the other two models). For both of these figures, we have ordered the plots in terms of the minimum number of observations removed, so, as one moves down the rows and across the columns, a greater number of observations have been removed. Each point on these plots presents the coefficient for one universe in our multiverse analysis.

Examining Figs. 2 and 3, a number of important points stand out. First, it appears that several of the panels present identical information. To check this, we compared the base datasets with one another. These comparisons revealed that the No Cleaning dataset was identical to the Stage 3 Off dataset, the Stage 4 Off dataset, and the Stage 4 Min = 80, Max = 800. The remaining datasets were not identical to one another. Overall, however, there was no clear pattern that emerged which clearly drove the magnitude of the observed frequency effects.

Second, although the base datasets might remove differing numbers of observations, and so the points and lines on the panels have different starting points on the *x* axes, the general pattern is that as the number of observations removed increases, the frequency effect coefficient reduces. This is consistent with the observations made by Eskenazi (2024): by removing progressively more data points, the frequency effect does not disappear, but is instead reduced in size.

## Discussion

The process of cleaning and analyzing datasets from eye movement and reading experiments involves a series of decisions that researchers must make. As we have illustrated here, the number of choices for which multiple options are considered acceptable practice is very high indeed, and results in a very high level of researcher degrees of freedom (Eskenazi, 2024; Simmons et al., 2011). Understanding the consequences of these choices is important, particularly when seeking to ensure that a given effect is stable regardless of how the data are processed, cleaned, and analyzed. In a worst-case scenario, there is an outside possibility that effects are *created* by specific combinations of cleaning and analytic procedures but do not otherwise, in reality, exist. There is uncertainty in the field of eye movements during reading regarding where our practices for data cleaning come from. More importantly, some of these practices, though widely used, appear to have never been examined or validated in any published work that we know of. It is, therefore, anything but a foregone conclusion in terms of what the outcome of these cleaning procedures might be. In the current paper, we used a multiverse analysis to explore the impact of various decisions in cleaning and analyzing on the reported size of what is considered one of the most robust effects in eye movements during reading: the frequency effect (e.g., see Inhoff & Rayner, 1986).

The first conclusion that can be derived from our findings is that, as expected, the frequency effect during reading is very robust. Setting aside the 12 models that did not converge, the frequency effect was significant in *all* remaining models. In other words, the robustness of the frequency effect is such that it remained present through any combination of (reasonable and defensible) choices in the pipeline for cleaning and analyzing eye movement data. Within our laboratory, when training new researchers, we often teach them that if a study they have conducted does not replicate the frequency effect, the problem likely does not lie in the frequency effect not being real, but instead will have another cause (e.g., programming or analytic errors). We believe the current findings corroborate this approach.

Since the frequency effect was significant in virtually all of the models that we ran, our examination can move from asking what steps in the analytic pipeline can cause the frequency effect to become significant or nonsignificant to a more nuanced question: what steps in the analytic pipeline cause the frequency effect to increase or decrease in size? This is an important question to address, because we found that the magnitude of the frequency effect was quite variable, ranging from 10.54 ms to 16.57 ms (these values include frequency effects that have been back-transformed from the log-transformed models). This means that a substantial 36% of the size of the effect was due to the specific choices made during cleaning and analyzing the eye movement data. We will now describe how the approaches taken through each of the decision points influenced the size of the frequency effect.

### Decision point #1: Merging of fixations and temporal cutoffs

The different settings of the cleaning functions of Data Viewer had minimal impact on the size of the frequency effect. Indeed, four of the datasets were identical to one another. As we can see from Fig. 1, the different settings that we used for the cleaning function produced frequency effects across the range of available outcomes. As discussed in the Introduction, the specific values used in the elaborate stages of merging fixations and removing fixations based on temporal cutoffs appear to have been chosen and used extensively despite not having widespread empirical evidence of their benefits of efficacy. The lack of impact of the settings in the cleaning function, or indeed the lack of difference with the dataset where no cleaning happened, is therefore reassuring.

### Decision point #2: Removing trials with few fixations

Likewise, the removal of trials in which an unusually low number of fixations were made during the reading of a sentence had little impact on the reported size of the frequency effect in our analyses. However, this could be because the number of trials that were removed for these reasons was fairly low. In the perhaps most “raw” of the datasets that we used, the No Cleaning dataset, only 24 trials out of 23,400 had fewer than three fixations (0.17%), and only 60 trials had fewer than five fixations (0.25%).

### Decision point #3: Removal of outlier fixations

The choice to compare fixation durations to a computed grand mean, the mean of a specific subject, or the mean of a specific subject in a specific condition did have a clear influence on the size of the frequency effect. As shown in Fig. 1, the largest frequency effects here were observed when trimming on the basis of the mean of a specific subject in a specific condition. Next, we have trimming by participants, and finally, trimming by the grand mean seems to, in many cases, produce slightly smaller frequency effects. The choice between removing as outliers fixations that were 2.5 versus 3 standard deviations from a given mean appeared to make very little difference to the size of the frequency effect.

### Decision point #4: Linear mixed effects models

As far as assumptions on the distributions are concerned, we found the largest frequency effects when a linear mixed model was run on the untransformed data assuming a normal distribution, and considerably smaller when there was no assumption made about the data being normally distributed. After back-transforming the reported sizes of the frequency effect of the linear mixed models that were run on the log-transformed data, this approach leads to an intermediate position in terms of the size of the effect between the two other approaches.

### Implications beyond the frequency effect in eye movements and reading

The variation in outcomes for the size of the frequency effect is important to consider. Though this variation did not influence the outcome of the analyses here in terms of significant frequency effects, this is likely to be the case for other effects in the field. Given the observed impact of specific choices made during cleaning and analysis on the size of the frequency effect, it is reasonable to assume that for weaker (or controversial) effects, these choices—though all considered reasonable practices—could determine whether a statistically significant effect is observed or not.

As shown in Figs. 2 and 3, the global rule seems to be that the more data are trimmed, the smaller the reported size of the frequency effect becomes. This is also compatible with the findings from Eskenazi (2024), who created three data sets, one with no data cleaning, one data set with a somewhat average amount of data removed, and one dataset with very strict exclusion criteria leading to a sizeable amount of data removal. He observed that standardized estimates decreased for the frequency effect as more data were removed. However, so did the variance in the data set; therefore, effects typically remained statistically significant, as they did in our study. We significantly expanded on this study by including more cleaning decisions, by also examining assumptions about the underlying distribution of the fixation duration data and its impact on how the linear mixed models are implemented, and by running a more powerful multiverse analysis instead of comparing three datasets. However, our conclusions are very much in line with those made by Eskenazi (2024).

Another consideration is that the different choices in cleaning methods will impact outlier removal and, as such, might mostly impact the tail of the distribution. Staub et al. (2010) carried out an ex-Gaussian analyses on eye movement during reading, comparing distributions of fixations on high- versus low-frequency words. They observed that the frequency manipulation influenced both the mean and the skew of the distributions. An interesting idea for future multiverse analysis could be to examine the impact of cleaning and analysis methods on an effect that exclusively impacts the means of distributions, as the impact of choices might be reduced for such an effect.

### Recommendations for data cleaning and analysis?

Given our approach here, it would be tempting to assume that we can create a set of recommended “best practices” for data cleaning and analysis in studies of this type. However, our goal was not to develop a list of recommendations regarding how to clean and analyze datasets from eye movement and reading experiments. What the multiverse analysis we have conducted has shown, as have other such multiverse analyses, is that it is difficult to predict the consequences of data processing and analytic pipelines in advance. Instead, because, for example, the cleaning that takes place at a given stage depends heavily on the cleaning that took place at previous stages in the process, one cannot readily discern how a given route through the garden of forking paths might proceed. Indeed, we cannot formulate strong reasons why the temporal cutoff for fixations should be such that fixations shorter than 80 ms should be removed as opposed to those shorter than 100 ms. However, the fact that these types of cleaning decisions seem to have minimal impact is reassuring. All we can recommend for now is that researchers in our field consider, test, and check the consequences of their cleaning and analytic decisions. Moreover, we recommend that the reasoning behind such decisions be made as transparent as possible, highlighting any supporting evidence of prior experimentation or empirical validation of cleaning procedures, where such evidence exists. Where no such evidence is known to exist, we recommend honesty and transparency should values for outliers and other cleaning procedures be determined by the individual decisions of researchers.

One could argue that, given our consistent findings that the frequency effect emerged regardless of the cleaning procedures that were adopted, the optimal approach for the field would be to adopt a “no cleaning” policy for datasets derived from eye movement and reading experiments. This would be dangerous, in our view, and for several reasons. First, our evidence here rests on a single study testing a single dependent variable examining a single effect in eye movements and reading. There are many other studies, dependent variables, and effects that would need to be examined carefully before making any such recommendations for changes to current procedures. Second, though there may be disagreement regarding the source of some of the cleaning procedures used in the field, the cleaning procedures are all ultimately based upon logical assumptions regarding the need to remove erroneous datapoints that emerge due to either participant or equipment error.

Third, there is a fundamental issue when cleaning datasets, in that it is difficult to “prove,” for example, that during any given fixation a participant made an error, or that there was an equipment error which resulted in an erroneous recording of the fixation. The only evidence available when deciding whether a fixation duration was an outlier is on the basis of whether it was too long or too short compared to some other criteria that we set. Given this intrinsic difficulty in being certain that any fixation is an outlier, it seems sensible to adopt a conservative approach and permit only the fixations that we do not doubt into our analyses. For that reason, in particular, taking a “no cleaning” approach would inevitably permit fixations that arose from participant or equipment error into our analysis, which would, of course, be counterproductive. This is because, primarily, the data points that we would want to include in our analyses are those in which participants are attentively and accurately engaging in the task, and those which have been veridically recorded by the eye tracker.

That being said, it is concerning that we have, here, also highlighted a *scientific* or *academic urban legend* (e.g., see Rekdal, 2014) regarding why the cleaning functions offered by SR Research’s Data Viewer software are used by many researchers to clean and process their data from eye movement and reading experiments (as also discussed by Eskenazi, 2024). It seems there have been no historic formal attempts directed at empirically validating each stage of the cleaning pipeline. Instead, researchers have simply adopted a convention for cleaning their data in a similar manner and assumed that the stages have been validated by others. Scenarios of this nature are not uncommon, unfortunately (Flake & Fried, 2020), and this, together with our overall finding that the cleaning function settings did not influence how the frequency effect emerged, means that the cleaning stages used by researchers in our field require further detailed scrutiny.

### Future work

We chose the frequency effect as the first candidate for a multiverse analysis of the impact of choices of cleaning and analysis as it allows us to examine how the reported size of a well-established effect is influenced. In future work, we plan on moving to examine other effects from the literature that are less clear-cut than the frequency effect. In particular, we plan next to conduct multiverse analyses of the parafoveal preview effect. The parafoveal preview effect is the finding that fixation times on a word are shorter when the preview of the word before the eyes landed on it was correct compared to when it was masked (Rayner, 1975). This effect demonstrates how, during reading, information from the next word, which is typically located in the parafovea, is processed even before the eyes land on it. This preview benefit is usually established by means of the eye-contingent boundary paradigm (Rayner, 1975). The preview of a word is either correct or masked until the eyes cross an invisible boundary in the text. When the tracker detects the eyes having passed the boundary, a quick display change is executed, replacing the masked preview with the correct word. Because the display change happens during a saccade, whilst the eyes are functionally blind, participants are usually unaware of this display change. A number of decisions are being made whilst cleaning up the eye movement data in this paradigm, such as setting the criterion for how many samples are allowed on the word after the boundary before the display change has actually happened. A too high number will mean the participants might be processing the mask in foveal vision and the trial will need to be removed. Setting this criterion to very few samples past the boundary would result in too many trials being thrown away. A multiverse analysis exploring decisions in this paradigm, such as the number of samples past the boundary resulting in the removal of the trial, would be able to give a definitive answer on what the best settings would be. Likewise, a multiverse analysis could be employed to examine the circumstances under which so-called *parafoveal-on-foveal effects* are observed. Parafoveal-on-foveal effects occur when the fixation time on a word is influenced by characteristics of the next word, which will typically be located in the parafovea. The existence of these effects is controversial (Brothers et al., 2017; Drieghe, 2011) and might be influenced by differences in the applied cleaning and analyses pipelines.

### Limitations

Conducting the multiverse analysis required running close to a million linear mixed models. This was not a small endeavor and necessitated making choices that will somewhat limit the applicability of the current findings. For starters, even though the literature search reported by Eskenazi (2024) indicated that in relatively recent papers, 96% of studies that reported eye movement tracking during reading used an EyeLink, other trackers are still being used (e.g., Fourward Technologies Dual-Purkinje Eyetracker). Perhaps more importantly, even though many researchers who use an EyeLink will use the Data Viewer software which has the clean function we examined, other researchers do use other software, such as MATLAB, which will allow for different processing pipelines. A second limitation we already mentioned is that we examined eye movement behavior during the reading of sentences. Paragraph reading comes with added complexities related to which line a fixation will be assigned to, and merits separate investigation. Thirdly, our mixed linear models were intercept-only, whereas research has pointed to the necessity of having a maximal (or more often as maximal as possible) random structure (Barr et al., 2013). We suspect that multiverse analyses will be ideally suited for future research to examine the consequences of trimming strategies, and the arrival of packages such as *buildmer* (Voeten, 2023) allow for different strategies to be implemented automatically. Finally, even though the frequency effect was the ideal candidate in which to observe the impact of different cleaning and analysis pipelines on the size of a well-established effect, this choice also came with limitations. Ex-Gaussian analyses have shown that frequency affects both the mean and the skew of the fixations distributions (Staub et al., 2010), raising questions on what our results would look like for an effect that exclusively impacts either the mean or the skew of the fixation time distribution.

### Summary and concluding comments

To summarize, we used a multiverse analysis to examine the impact of choices made during the cleaning and analysis of eye movements during reading on the reported size of the frequency effect. We observed that some decisions seem to have minimal impact on the size of the frequency effect, such as decisions on merging fixations and which temporal cutoffs are used, or the decision to remove trials based on an unusually low number of fixations. However, the decision on which assumptions are made about the underlying distribution of the fixation data and its impact on how the linear mixed models are implemented does have a substantial impact, with the largest frequency effect observed when untransformed fixation data are analyzed in a linear mixed model that assumes a normal distribution. Likewise, when removing outliers based on standard deviations away from the mean, the choice of which mean is used influences the reported size of the frequency effect: the smallest effect is reported when using the grand mean, a larger effect when using the mean for a specific subject, and the largest effect when using the mean for a specific subject in a specific condition. The combined impact of these choices could lead to a frequency effect that is 36% greater than the smallest observed effect, even though we restricted ourselves to exploring choices that are considered reasonable. One of the goals of this paper was to raise awareness of the impact of the choices researchers make whilst cleaning and analyzing eye movement when reading data and to also introduce multiverse analyses to the research field. Although this paper is not intended to be a tutorial on running these types of analyses, we have made our code available and have made a strong effort to add comments in the code as much as possible, therefore making it feasible to adjust for other experiments. We refer the interested reader to a range of available tutorials (Del Giudice & Gangestad, 2021; Simonsohn et al., 2020) for further information.

## Data Availability

All code and data relating to the secondary data analyses reported here are available at: https://osf.io/2d3um/?view_only=30a9ba7f41164732b37ed700b3f2dab8.

## Footnotes

1 An area of interest is a virtual “box” drawn around words in reading experiments. When a fixation lands within an area of interest, it is recorded that the participant was fixating on the word contained within that interest area.

## Declarations

### Open practice statement

The raw data files and R scripts used for the statistical analyses are publicly available at https://osf.io/2d3um/?view_only=30a9ba7f41164732b37ed700b3f2dab8. This study was not pre-registered.
